# Application of Petri Nets in Bone Remodeling

**DOI:** 10.4137/grsb.s2881

**Published:** 2009-07-06

**Authors:** Lingxi Li, Hiroki Yokota

**Affiliations:** 1 Departments of Electrical and Computer Engineering and; 2 Biomedical Engineering Indiana University—Purdue University Indianapolis, Indianapolis, IN 46202. Email:hyokota@iupui.edu

**Keywords:** Petri nets, bone remodeling, metabolic networks, invariant analysis

## Abstract

Understanding a mechanism of bone remodeling is a challenging task for both life scientists and model builders, since this highly interactive and nonlinear process can seldom be grasped by simple intuition. A set of ordinary differential equations (ODEs) have been built for simulating bone formation as well as bone resorption. Although solving ODEs numerically can provide useful predictions for dynamical behaviors in a continuous time frame, an actual bone remodeling process in living tissues is driven by discrete events of molecular and cellular interactions. Thus, an event-driven tool such as Petri nets (PNs), which may dynamically and graphically mimic individual molecular collisions or cellular interactions, seems to augment the existing ODE-based systems analysis. Here, we applied PNs to expand the ODE-based approach and examined discrete, dynamical behaviors of key regulatory molecules and bone cells. PNs have been used in many engineering areas, but their application to biological systems needs to be explored. Our PN model was based on 8 ODEs that described an osteoprotegerin linked molecular pathway consisting of 4 types of bone cells. The models allowed us to conduct both qualitative and quantitative evaluations and evaluate homeostatic equilibrium states. The results support that application of PN models assists understanding of an event-driven bone remodeling mechanism using PN-specific procedures such as places, transitions, and firings.

## Introduction

Bone remodeling is a homeostatic process for maintenance of healthy bone through a removal of old bone by osteoclasts followed by deposition of new bone by osteoblasts.^1^ Since the process is a complex interplay among many molecules and varying types of cells, building a mathematical model is useful for developing therapeutic strategies for patients with metabolic disorders such as osteoporosis. Pioneering modeling works using a set of ordinary differential equations (ODEs) includes evaluation of the effects of parathyroid hormone (PTH) and the role of a signaling system composed of osteoprotegerin (OPG), receptor activator of nuclear factor κB (RANK), and RANK ligand (RANKL).^2^–^4^ Although describing a bone remodeling process using ODEs is a basis for quantitative analyses, numerical results often present a challenge in interpreting the role of discrete molecular and cellular events involved in bone remodeling.

Here, we applied Petri nets (PNs) as an alternative approach to simulate interactive events in bone remodeling. Compared to the approach with ODEs, PNs offer several advantages. First, PNs provide graphical representation of individual interactions in the system that seems appropriate for modeling, analysis and simulation of large-scale dynamic systems.^5^–^7^ Second, since system behaviors in PNs are monitored by discrete events through the firing of transitions, PNs can offer a framework for implementing complex temporal inter-related events (both synchronous and asynchronous) as well as structural interactions. Third, since any events are generated and transformed in the network model, not only deterministic but also stochastic processes can easily be built in. Fourth, PNs enable us to conduct both qualitative systems analysis (structural characterization) and quantitative analysis (monitoring dynamic behaviors).

PNs have been extensively applied in many engineering areas such as manufacturing systems,^8^–^10^ transportation systems,^11^,^12^ and communication networks.^13^,^14^ Recently, PNs have been applied for modeling and analysis of metabolic pathways. For instance, qualitative analyses have been conducted focusing on place invariants and transitions invariants^15^,^16^ as well as steady states of metabolic pathways.^17^,^18^ Quantitative analyses have also been performed in calculation of the probability distribution of molecular species^19^–^21^ and molecular concentrations.^22^–^24^ Few studies, however, have been directed to both qualitative and quantitative analyses with reference to the ODE-based approach. Our particular interest herein is to evaluate ODE-driven equilibrium states using a PN model. This evaluation is especially important for physiological processes like bone remodeling, where variations from homeostatic equilibria may be linked to metabolic disorders.

In order to examine a potential capability of PNs in bone remodeling, it is neither feasible nor desirable to attempt to build models that include many unknown factors. We thus focused on one of the major signaling pathways (OPG-RANK-RANKL pathway) with four dominant bone cell types (two types of osteoblasts and two types of osteoclasts). Using PN models, our qualitative analysis was focused on identifying two properties (place invariants and transition invariants). Place invariants are for characterizing relationships among variables, while transition invariants are for identifying a set of sub-networks in the overall network. In the quantitative analysis, we evaluated the homeostatic equilibrium states based on PNs and compared them with the results obtained from ODEs.

## Methods

### Derivation of ODEs

**Figure 1:**
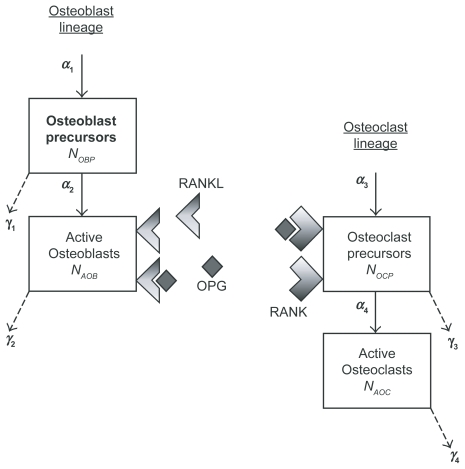
Schematic illustration of bone remodeling focusing on interactions among osteoblasts and osteoclasts through OPG/RANK/RANKL pathway.

The bone remodeling process was modeled using 8 state variables (4 in the molecular level, and 4 in the cellular level) (Fig. 1). In the molecular level, 4 state variables focusing on OPG/RANK/RANKL pathway were *x_O_*(*t*), *x_L_*(*t*), *x_OL_*(*t*), *x_KL_*(*t*), which corresponded to the concentrations of OPG (O), RANKL (L), RANK (K), OPG-RANKL complex (OL), and RANK-RANKL complex (KL). The first-order ODEs were (*ẋ*= time derivative of state *x; k_i_* = rate constant; *p_i_* = synthesis rate; and *d_i_* = degradation rate):

(1) $${\dot{x}}_{O}(t)=p_{O}-k_{1}x_{O}(t)x_{L}(t)+k_{2}x_{OL}(t)-d_{O}x_{O}(t)$$

(2) $$\begin{array}{l} {\dot{x}}_{L}(t)=p_{L}-k_{1}x_{O}(t)x_{L}(t)+k_{2}x_{OL}(t)\\ -k_{3}x_{K}(t)x_{L}(t)+k_{4}x_{KL}(t)-d_{L}x_{L}(t) \end{array}$$

(3) $${\dot{x}}_{OL}(t)=k_{1}x_{O}(t)x_{L}(t)-k_{2}x_{OL}(t)$$

(4) $${\dot{x}}_{KL}(t)=k_{3}x_{K}(t)x_{L}(t)-k_{4}x_{KL}(t)$$

In the cellular level, 4 state variables represented the numbers of 4 different types of cells (OBP = osteoblast precursors; AOB = active osteoblasts; OCP = osteoclast precursors; and AOC = active osteoclasts). Amplification and differentiation of those cells were modeled:

(5) $${\dot{N}}_{OBP}(t)=\alpha_{1}-\gamma_{1}N_{OBP}(t)$$

(6) $${\dot{N}}_{AOB}(t)=\alpha_{2}N_{OBP}(t)+\beta_{2}x_{OL}(t)-\gamma_{2}N_{AOB}(t)$$

(7) $${\dot{N}}_{OCP}(t)=\alpha_{3}+\beta_{3}x_{KL}(t)-\gamma_{3}N_{OCP}(t)$$

(8) $${\dot{N}}_{AOC}(t)=\alpha_{4}N_{OCP}(t)-\gamma_{4}N_{AOC}(t)$$

where *N* = number of cells; *α_i_* = synthesis rate; *β_i_* = interaction factor, and *γ_i_* = degradation rate. The predicted values of the above parameters, employed in this study, are listed in Table 1.

### Identification of equilibrium states

Although the equilibrium state values vary depending on the parameter values, there is only one equilibrium condition in Eqs. (1–8), where all time derivatives = 0. Their values were analytically derived: *x_O_^EQU^* = 5.71 × 10^2^ nM; *x_L_^EQU^* = 2.86 × 10^0^ nM; *x_OL_^EQU^* = 1.63 × 10^3^ nM; *x_KL_^EQU^* = 8.57 × 10^1^ nM; *N_OBP_^EQU^* = 80,000; *N_AOB_^EQU^* = 40; *N_OCP_^EQU^* = 9.0; and *N_AOC_^EQU^* = 0.90. Note that the unit for the cell numbers was chosen arbitrary.

### Modeling strategy with PNs

**Figure 2:**
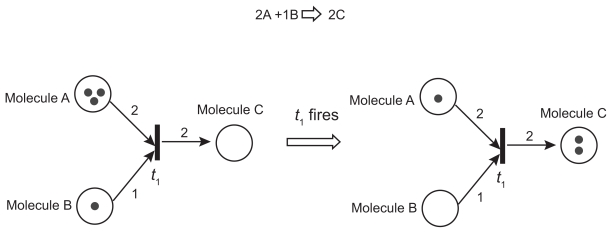
Simplified PN model for a molecule synthesis process from molecules A and B to molecule C.

To illustrate our modeling strategy, a simplified version of PN models is shown (Fig. 2). PNs are weighted bipartite graphs with two types of nodes (places and transitions) and arcs. Places (circles in Fig. 2; e.g. molecules and cells) indicate the conditions under which transitions can occur, and transitions (bars in Fig. 2) mark events that alter states (e.g. synthesis, degradation, and chemical reaction). Arcs (arrows in Fig. 2) capture casual relationships as well as interactions among nodes, and they are associated with integer weights that regulate events. The states of PNs are defined by tokens (black dots inside places in Fig. 2), which represent the number of resources (e.g. number of molecules). In the example in Fig. 2, two molecules A and one molecule B are required to synthesize two molecules C, and this event is regulated by the firing of the transition *t*_1_.

### Qualitative PN analysis

In qualitative PN analysis, two behavioral properties (place invariants and transition invariants) were examined. Place invariants are a set of places where the number of tokens in those places remains constant during the evolution (dynamic behaviors) of the system. They identify the processes in which the numbers of molecules or cells stay unchanged. Transition invariants are a set of transitions where their sequences of firings can be reproduced in the specific states. They are useful to capture cyclic reaction processes and can be used to identify reversible subnetworks in metabolic networks.

### Quantitative PN analysis

PNs represent discrete state and event-driven systems, and quantitative PN analysis was conducted using numerical integration. In our analysis, the dynamic behaviors in PNs were characterized using a flow of tokens triggered by firings of transitions. The regulatory rules were derived for each firing of transitions from the parameters in ODEs, and tokens were added or removed based on the network structures. Note that since ODEs offer continuous quantities, those continuous quantities were discretized in the simulation step (in terms of the number of tokens in places). Using a set of initial conditions, we traced the numbers of tokens in the places and evaluated their temporal alterations with reference to the equilibrium states derived from ODEs. Although the time axis was defined in terms of the event-driven firing sequences, it was uniquely linked to real time.

## Results and Discussion

### PN model

**Figure 3:**
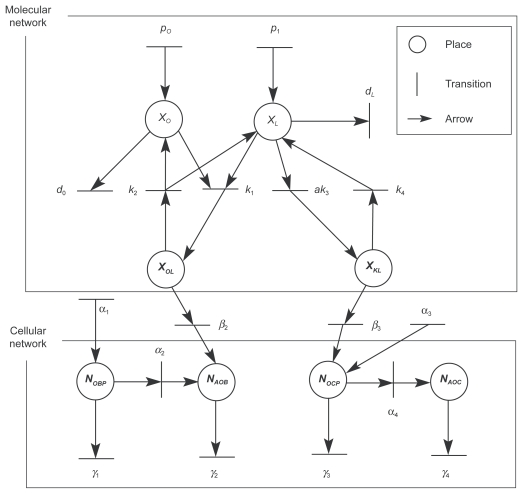
PN model for bone remodeling including a molecular network and a cellular network.

The overall PN model for the selected bone remodeling process is illustrated (Fig. 3). In this model, 8 state variables were considered including 4 variables in the molecular sub-network (concentrations of OPG, RANKL, OPG-RANKL complex, and RANK-RANKL complex) and 4 variables in the cellular sub-network (numbers of osteoblast precursors, active osteoblasts, osteoclast precursors, and active osteoclasts). Eight places (circles) designated these state variables, and interactions and dependencies among them were represented by transitions (bars) derived from Equations (1–8). The entire PN network included 4 chemical rate constants (*k*_1_ – *k*_4_), 2 molecular synthesis rates (*p_O_* and *p_L_*), 2 molecular degradation rates (*d_O_* and *d_L_*), 4 cellular synthesis rates (*α*_1_ – *α*_4_), 4 cellular degradation rates (*γ*_1_ – *γ*_4_), and 2 molecular/cellular interaction factor (*β*_2_, *β*_3_). The two sub-networks were connected through the arrows with (*β*_2_, *β*_3_), and *x_k_*(*t*) was set to constant as “*a*”.

Prior to qualitative and quantitative analyses, we evaluated the sensitivity of the equilibrium states to the selected parameters. We first obtain the equilibrium states analytically from the set of ODEs:

$$\begin{array}{l} x_{O}^{EQU}=p_{O}/d_{O};x_{L}^{EQU}=p_{L}/d_{L};\\ x_{OL}^{EQU}=(k_{1}/k_{2})(p_{O}/d_{O})(p_{L}/d_{L});\\ x_{KL}^{EQU}=(k_{3}/k_{4})(p_{L}/d_{L})x_{K};N_{OBP}^{EQU}=\alpha_{1}/\gamma_{1};\\ N_{AOB}^{EQU}=\alpha_{1}\alpha_{2}/\gamma_{1}\gamma_{2}+(\beta_{2}k_{1}/\gamma_{2}k_{2})(p_{O}/d_{O})(p_{L}/d_{L});\\ N_{OCP}^{EQU}=\alpha_{3}/\gamma_{3}+(\beta_{3}k_{3}/\gamma_{3}k_{4})(p_{L}/d_{L})x_{k};\\ N_{AOC}^{EQU}=\alpha_{3}\alpha_{4}/\gamma_{3}\gamma_{4}+(\alpha_{4}\beta_{3}k_{3}/\gamma_{3}\gamma_{4}k_{4})(p_{L}/d_{L})x_{k}. \end{array}$$

Then a partial derivative of all equilibrium states was derived with respect to each of the chosen parameters such as ∂*x_O_*/∂*p_O_*; ∂*x_O_*/∂*d_O_,* …, ∂*N_AOC_*/∂*γ*. There were 144 derivatives corresponding to 8 state variables and 18 parameters, and 43 derivatives were non zero. Two parameters (*p_L_* and *d_L_*) were involved in the equilibrium states of 6 state variables, while 5 parameters (*α*_2_, *α*_4_, *β*_2_, *γ*_2_, *γ*_4_) were linked to a single equilibrium state only. Among 6 state variables affected by *p_L_*, for instance, the most sensitive state to a variation of *p_L_* value was *x_OL_*.

### Qualitative analysis

The structural PN model in Fig. 3 did not have any place invariants. This result implies that no conservation of molecules or cells that were involved in this metabolic process. However, 11 transition invariants were identified (Table 2). For instance, the invariant sub-network *p_O_* → *p*_1_ → *k*_1_ → *β*_2_ → *γ*_2_ corresponds to a process of the synthesis of OPG-RANKL complexes from OPG and RANKL and its interaction with active osteoblasts, while the invariant sub-network *p*_1_ → *ak*_3_ → *β*_3_ → *α*_4_ → *γ*_4_ corresponds to the interactions among RANKL, RANK-RANKL complex, osteoclast precursors, and active osteoclasts. Furthermore, the sub-networks *k*_2_ → *k*_1_ and *ak*_3_ → *k*_4_ corresponds to the reversible processes among OPG, RANKL and OPG-RANKL complexes, and between RANKL and RANK/RANL complexes, respectively.

### Quantitative analysis

#### Simulation of a sub-network I

**Figure 4:**
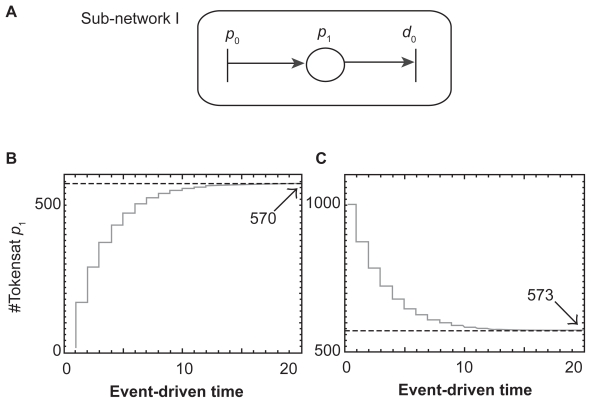
Simulation of sub-network I. **A**) Sub-network I with a single place, *p*_1_. **B**) Transient response of the token number in the place *p*_1_ starting with the initial concentration of 5 nM. **C**) Transient response of the token number in the place *p*_1_ starting with the initial concentration of 1000 nM.

We first examined the transient responses of a simplified PN model (sub-network I) (Fig. 4). In this sub-network, the concentration of OPG, *x_O_*(*t*), assigned in place *p*_1_, was expressed in ODE: *ẋ_O_*(*t*) = *p_O_* − *d_O_* *x_O_* (*t*). In our numerical PN simulation, we set *p_O_* = 200 nM/day and *d_O_* = 0.35/day with two initial OPG concentrations at 5 nM and 1000 nM. The results revealed that regardless of the initial OPG concentration its steady-state concentration approached to the ODE predicted equilibrium (steady-state) value at *p_O_*/*d_O_* (200/0.35 = 571.4 nM).

#### Simulation of the sub-networks I and II

**Figure 5:**
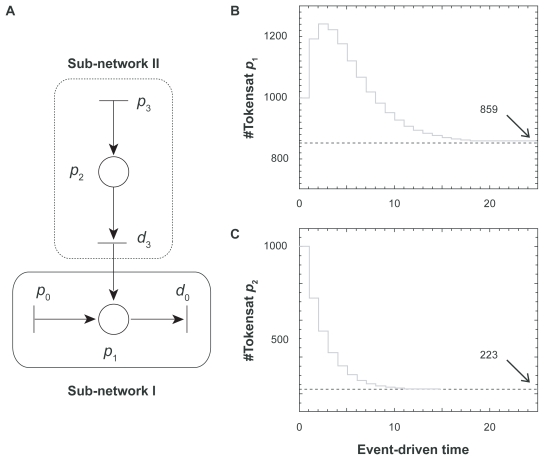
Simulation of sub-networks I and II. **A**) Sub-networks I and II including two states places, *p*_1_ and *p*_2_. **B**) Transient response of the token number in the place *p*_1_. **C**) Transient response of the token number in the place *p*_2_.

We next evaluated the interaction between two sub-networks, which were described in ODEs as: *ẋ_O_*(*t*) = *p_O_* + *d*_3_*x*_1_(*t*) − *d_O_x_O_*(*t*) and *ẋ*_1_(*t*) = *p*_3_ − *d*_3_*x*_1_(*t*), where *x_O_*(*t*) = state variable denoted by place *p*_1_, and *x*_1_(*t*) = state variable denoted by place *p*_2_ (Fig. 5). The parameters for PN simulations were: *p_O_* = 200 nM/day, *d_O_* = 0.35/day, *p*_3_ = 100 nM/day, and *d*_3_ = 0.45/day. Starting with the initial concentrations of 1000 nM at both places *p*_1_ and *p*_2_, the transient responses (alterations in the numbers of tokens) in the places *p*_1_ and *p*_2_ are plotted. Because of the interactions between the two sub-networks I and II, the equilibrium states of *x_O_*(*t*) was different from the result in the sub-network I alone. In concert to the ODE-based predictions [(*p_O_* + *p*_3_)/*d_O_* = (200 + 100)/0.35 = 857.1 nM for *x_O_*(*t*) and *p*_3_/*d*_3_ = 222.2 nM for *x*_1_(*t*)], our PN results offered 859 and 223 for *x*_0_ and *x*_1_, respectively.

#### Evaluation of the equilibrium states using the entire PN model

**Figure 6:**
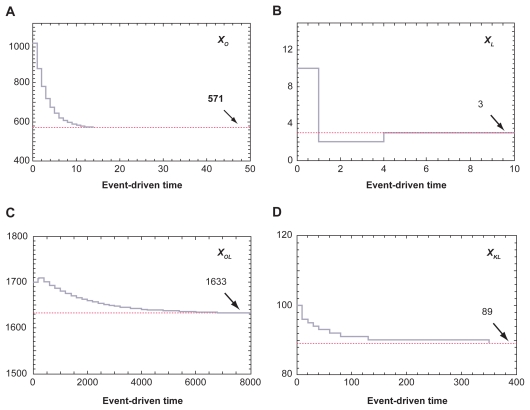
Evolution of 4 state variables in the molecular network towards ODE-predicted equilibrium states. **A**) Evolution of *x_O_*(*t*). **B**) Evolution of *x_L_*(*t*). **C**) Evolution of *x_OL_*(*t*). **D**) Evolution of *x_KL_*(*t*).

**Figure 7:**
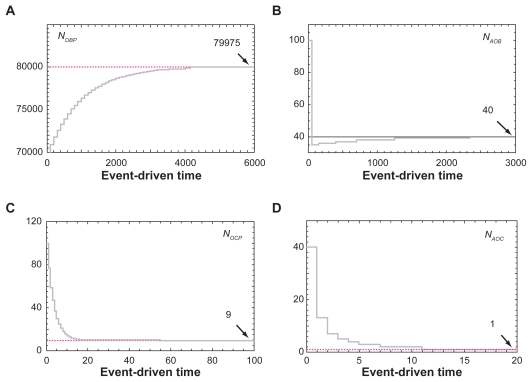
Evolution of 4 state variables in the cellular network towards ODE-predicted equilibrium states. **A**) Evolution of *N_OBP_*(*t*). **B**) Evolution of *N_AOB_*(*t*). **C**) Evolution of *N_OCP_*(*t*). **D**) Evolution of *N_AOC_*(*t*).

The transient responses for the entire PN model were simulated using the initial conditions that deviated from the ODE predicted equilibrium states. Although time required for reaching steady states varied among 8 state variables, all 8 variables returned closely to the ODE equilibrium states (Figs. 6 and 7). First, the results for the molecular network in Fig. 6 revealed that the steady state PN values were 571, 3, 1633, and 89 nM for *x_O_*, *x_L_*, *x_OL_* and *x_KL_*, respectively. The ODE predicted values were 571, 2.86, 1630, and 85.7 nM in this order. Second, the steady state PN values for the cellular network in Figure 7 exhibited 79,975 for *N_OBP_*, 40 for *N_AOB_*, 9 for *N_OCP_*, and 1 for *N_AOC_*, while the ODE predictions were 80,000, 40, 9.0, and 0.9 respectively.

In the current study, we conducted both qualitative and quantitative analyses. Our qualitative analysis allowed us to identify reversible processes, and determine interactions and dependencies among molecules and cells. The quantitative analysis for equilibrium states enabled to establish a bridge between the ODE-based continuous responses and the event-driven discrete networks. The potential capability of PNs in investigating metabolic networks is multifold. First, unlike ODEs PN models can easily incorporate non-differentiable functions. For instance, administration of therapeutic agents such as OPG can be given in an arbitrary form including a series of impulsive dosages. Second, the potential effect of individual molecules such as RANK can be monitored graphically in any sub-networks. Third, an effect of a single event (e.g. synthesis of one molecule) in the entire PNs can be evaluated. Fourth, differential transient responses and time constants can be determined through temporal evolutions among variables. Lastly, although the described bone remodeling model is much simpler than a true physiological phenomenon, the present PN model can be expanded by adding more places and transitions.

Since OPG can reduce bone resorption through interactions with RANK and RNAKL, it can be used as a therapeutic agent for patients with osteoporosis.^25^ In order to achieve a suitable outcome without inducing potential side effects, the administration sequence (timing and dosage) needs to be evaluated. We believe that the PN model in the current study can be used to predict a safe, effective administration strategy. Bone is a complex organ, and biological and mechanical characters differ depending on locations. Although the current PN model does not include those local variations, it is possible to differentiate site-specific dynamics by considering additional state variables and parameters.

## Conclusion

The study herein presented a unique PN model for evaluation of bone remodeling focusing on the OPG/RANK/RANKL signaling pathway among precursor/active osteoblasts and osteoclasts. The described PN model is able to characterize qualitative (structural) and quantitative (dynamic) properties of the complex homeostatic process. It identified transition invariants (closed-loops and reversible processes) and verified the equilibrium states derived from the associated set of ODEs. Since PN’s discrete network modeling fits well to event-driven physiological responses, further application of PN models should contribute to the understanding of complex molecular and cellular interactions and development of therapeutic strategies in bone remodeling and other biological processes.

## Figures and Tables

**Table 1:** Parameters and RANK concentration employed in equations (1–8).

Symbol	Value	Unit	Symbol	Value	Unit
**Chemical rates**			**Cellular proliferation rate**		
*k*_1_	10	1/(nM•day)	*α*_1_	80	1/day
*k*_2_	10	1/day	*α*_2_	0.001	1/day
*k*_3_	0.6	1/(nM•day)	*α*_3_	1	1/day
*k*_4_	0.02	1/day	*α*_4_	0.2	1/day
**Molecular synthesis rate**			**Factors for molecular/cellular interactions**		
*p_O_*	200	nM/day	*β*_2_	0	1/(nM•day)
*p_L_*	1	nM/day	*β*_3_	0.02	1/(nM•day)
**Molecular degradation rate**			**Cellular degradation rate**		
*d_O_*	0.35	1/day	*γ*_1_	0.001	1/day
*d_L_*	0.35	1/day	*γ*_2_	2	1/day
**RANK concentration**			*γ*_3_	0.3	1/day
*x_k_*	1	nM	*γ*_4_	2	1/day

**Table 2:** Summary of transition invariants in the PN model.

Transition invariants	Remarks
*p*_0_ → *p*_1_ → *k*_1_ → *β*_2_ → *γ*_2_	Synthesis of OPG-RANKL from OPG and RANKL, and its interaction with active osteoblasts
*p*_O_ → *d*_O_	Synthesis and degradation of OPG
*p*_1_ → *ak*_3_ → *β*_3_ → *γ*_3_	Closed-loop interactions among RANKL, RANK-RANKL, and osteoclast precursors
*p*_1_ → *ak*_3_ → *β*_3_ → *α*_4_ → *γ*_4_	Interactions among RANKL, RANK-RANKL, osteoclast precursors, and active osteoclasts
*ak*_3_ → *k*_4_	Reversible process between RANKL and RANK-RANKL
*k*_2_ → *k*_1_	Reversible process among OPG, RANKL, and OPG-RANKL
*p*_1_ → *d_L_*	Synthesis and degradation of RANKL
*α*_1_ → *α*_2_ → *γ*_2_	Interaction between osteoblast precursors and active osteoblasts
*α*_1_ → *γ*_1_	Synthesis and degradation of osteoblast precursors
*α*_3_ → *γ*_3_	Synthesis and degradation of osteoclast precursors
*α*_3_ → *α*_4_ → *γ*_4_	Interaction between osteoclast precursors and active osteoclasts
